# MicroRNA-30-3p Suppresses Inflammatory Factor-Induced Endothelial Cell Injury by Targeting TCF21

**DOI:** 10.1155/2019/1342190

**Published:** 2019-07-02

**Authors:** Zhenyu Zhou, Yu Chen, Dongying Zhang, Shiyong Wu, Tao Liu, Guoqiang Cai, Shu Qin

**Affiliations:** ^1^Department of Cardiology, The First Affiliated Hospital of Chongqing Medical University, Chongqing 400016, China; ^2^Department of Cardiology, Nanchong Central Hospital, The Second Clinical School of North Sichuan Medical College, Nanchong, China; ^3^Comprehensive Ward, Nanchong Central Hospital, The Second Clinical School of North Sichuan Medical College, Nanchong, China

## Abstract

Atherosclerosis is one of the leading causes of mortality worldwide. Growing evidence suggested that miRNAs contributed to the progression of atherosclerosis. miR-30-5p was found involved in various diseases. However, the role of miR-30-5p in regulation of atherosclerosis is not known. Here, we aim to investigate the effects of miR-30-5p on regulating the progression of atherosclerosis. The expression levels of miR-30-5p in serum collected from atherosclerosis patients and normal healthy people were analyzed by qRT-PCR. Gene Ontology (GO) and Kyoto Encyclopedia of Genes and Genomes (KEGG) pathway bioinformatics were carried out to reveal the possible signaling pathways involved in the mode of action of miR-30-5p. A potential target gene of miRNA-30-5p was searched and examined by a luciferase reporter assay. ELISA, Western blot, proliferation, and flow cytometry assays were performed to assess the biological functional role of miR-30-5p *in vitro*. Also, an *in vitro* monocyte-endothelial cell coculture model was used to study the functional role of miR-30-5p in atherosclerosis. We found that miR-30-5p was significantly decreased in serum samples from atherosclerosis patients compared with control subjects. GO and KEGG analysis results showed that miR-30-5p is highly associated with genetic profile of cardiovascular disease. *TCF21* was verified as a target gene of miR-30-5p. Overexpression of miR-30-5p in THP-1 not only protected endothelial cell viability but also inhibited endothelial cell apoptosis, and similar results were observed in cells with that of TCF21 knocked down. Moreover, miR-30-5p decreased the expression levels of lactate dehydrogenase (LDH) and tumor necrosis factor-*α* (TNF-*α*) and reduced reactive oxygen species (ROS) accumulation. NF-*κ*B and MAPK/p38 pathways played an indispensable role in the protection ability of miR-30-5p against atherosclerosis. Our results reveal that miR-30-5p suppresses the progression of atherosclerosis through targeting TCF21 *in vitro*. Therefore, the miR-30-5p-TCF21-MAPK/p38 signaling pathway may be a potential biomarker or therapeutic target in atherosclerosis.

## 1. Introduction

Atherosclerosis is a disease where the buildup of blood plaque causes the narrowing of arteries. Atherosclerosis and its complications such as coronary heart disease, carotid artery disease, and chronic kidney disease are still one of the major causes of mortality worldwide [1]. The molecular mechanism of atherosclerosis progressions has been studied for decades, and multiple pathways and physiopathological processes are involved in the development of atherosclerosis, such as inflammation, apoptosis, and necrosis as well as NF-*κ*B pathways and TLR4/MAPK pathways [2]. Treatments for atherosclerosis and its complications have been well developed and mainly focusing on drug treatment such as statins [3], PCSK9 inhibitors [4], and surgery on severe cases [5]. Due to the unsatisfactory therapeutic effect, the primary task to improve the treatment is to study the molecular pathogenesis and find more effective therapeutic targets of atherosclerosis. Recently, the functional roles of microRNAs in the pathogenesis of atherosclerosis and the development of advanced therapeutic strategies for atherosclerosis have been driven increasing attention.

MicroRNAs (miRNAs) are a class of short, endogenous noncoding single-stranded RNAs containing 21-25 nucleotides. They play critical regulatory roles in posttranscription in gene expressions by targeting specific mRNAs for destabilizing the mRNA, repressing protein production, and translational silencing [6]. With this powerful regulatory function, miRNAs contribute to various biological processes including proliferation [7], differentiation [8], and apoptosis [7]. Abundant evidences have showed that miRNAs are involved in pathogenesis of many diseases, especially tumor development [9]. However, in comparison to investigating miRNA in cancer development, the functional role of miRNAs in the pathogenesis of atherosclerosis is still undershadowed. Therefore, understanding the underlying molecular mechanisms of miRNAs in pathogenesis of atherosclerosis may contribute to develop advanced pharmacological therapeutic approaches for the treatment and prevention of atherosclerosis. Studies had reported that miR-21 [10], miR-126 [11], miR-145 [12], and miR-155 [13] contributed to the regulation of atherosclerosis by targeting PTEN, VCAM-1, KLF4, and MyD88, respectively. It has been found that miR-30-5p was downregulated in various human cancers [14], and upregulation of miR-30-5p decreases anti-inflammatory markers such as VCAM-1 and ICAM-1 by targeting angiopoietin-2 in endothelial cells [15]. However, a role for miR-30-5p in regulating endothelial inflammation and atherosclerosis needs to be investigated.

Ubiquitously expressed in numerous tissues [16], transcription factor 21 (TCF21) is shown to be important during embryogenesis of the heart [17]. Moreover, knockout of TCF21 was demonstrated as failure of cardiac fibroblast development [18], also suggesting the important role for TCF21 in the embryogenesis of the heart. In addition, evidences have suggested that TCF21 gene is required for cardiac fibroblast development [19], and the abnormal expression of TCF21 is associated with increased risk of coronary artery disease [20, 21]. On the other hand, TCF21 is found to be deregulated in various types of tumors and functions as a tumor suppressor [22]. However, whether miR-30-5p/TCF21 axis contributes to atherosclerosis is by far not clear.

The present study is aimed not only at investigating the effects of miR-30-5p on the progression of atherosclerosis *in vitro* but also at investigating the mechanisms that connected miR-30-5p/TCF21 and atherosclerosis. The findings reported in this study were of clinical significance due to the clarification of the impact of miR-30-5p/TCF21 on NF-*κ*B and MAPK/p38 signaling pathway in the THP-1/HUVEC *in vitro* model, and it could serve as a basis for the development of predictive biomarkers of atherosclerosis emergence and of preventive methods of atherosclerosis.

## 2. Materials and Methods

### 2.1. Patients and Sample Collection

Patients with atherosclerosis and without any treatments were involved in this study. Serum samples were collected from these patients. The control subjects were collected from normal people (volunteers). Interpretation of biopsy results was performed according to the Updated Banff 07 criteria by H.R. The protocols used in the present study are approved by Nanchong Central University. Written informed consents were obtained from all participants involved in the study.

### 2.2. Functional Annotation and Pathway Enrichment Analysis

GO analysis is an extraordinary useful method for annotating genes and is divided into three broad categories, namely, cellular component, molecular function, and biological process. The KEGG pathway database is a synthetic database, which includes a variety of biochemical pathways. In our study, the analyses of GO and KEGG pathway were performed with DAVID (Database for Annotation, Visualization, and Integrated Discovery, https://david.ncifcrf.gov/).

### 2.3. Cell Culture

Human umbilical vein cells (HUVECs) obtained from the Bena Culture Collection (Cat. No. ATCCPCS-100-010, ATCC, Manassas, VA, USA) were cultured in Endothelial Cell Medium (ScienCell, USA) with 5% (*v*/*v*) fetal bovine serum (FBS) (HyClone, South Logan, UT, USA), 100 U/ml penicillin, 100 mg/ml streptomycin (Invitrogen, Carlsbad, CA, USA), and 1% ECGF (Invitrogen). A human acute monocytic leukemia cell line (THP-1) was obtained from the National Infrastructure of Cell Line Resource (resource No. 3111C0001CCC000057), and then, the cell was cultured in RPMI-1640 medium (Gibco BRL) with 10% (*v*/*v*) FBS, 100 U/ml penicillin, and 100 mg/ml streptomycin. Cells were incubated in a 5% CO_2_ incubator at 37°C. At 70-80% confluence, cells were splitting according to standard procedures. THP-1 cells were harvested in RPMI-1640 medium treated with 50 *μ*g/ml ox-LDL (oxidized low-density lipoprotein) after being induced by 160 nM for 24 h. The *in vitro* atherosclerosis cell model was constructed using ox-LDL. For the coculture of THP-1 cells (monocytes) and human umbilical vein ECs (HUVECs), 5 × 10^5^ HUVEC cells were seeded into six-well plates, and then, 1 × 10^6^ ox-LDL-treated THP-1 cells were added onto the HUVEC layers. A transwell chamber was prepared for an indirect coculture.

### 2.4. Determination of Cell Proliferation by Cell Counting Kit 8 (CCK8)

Cell proliferation was evaluated by a cell counting kit 8 (CCK8) (Solarbio, Beijing, China) according to the manufacturer's protocol. Cells were cultured at a density of 5 × 10^4^ per well in a 96-well culture dish. After adherence, CCK8 solution was added to each well, and then, cells were further incubated for 2 hours at 37°C. The absorbance of samples at 450 nm was determined by a multiwell plate reader.

### 2.5. Assay of Apoptosis by Flow Cytometry

Apoptosis was determined by annexin V and propidium iodide (PI) double staining. After experimental treatment, cells were detached with trypsin-EDTA, washed twice with PBS, resuspended in binding buffer (10 mM HEPES pH 7.4, 150 mM NaCl, 5 mM KCl, 1 mM MgCl_2_, and 1.8 mM CaCl_2_) containing FITC-annexin V (1 g/ml), and then further incubated for 20 min. 10 minutes before the end of incubation, PI (10 g/ml) was added to this cell suspension in order to stain necrotic cells. Cells were analyzed with a FACScan flow cytometer equipped with an excitation laser line at 488 nm. The PI was collected through a 575 nm band pass filter.

### 2.6. ROS Detection

Cells were incubated with 10 *μ*M 2′,7′-dichlorofluorescin diacetate (DCFH-DA, Sigma-Aldrich, St. Louis, MO, USA) for 30 minutes, after which they were washed. ROS generation was determined by FACScan flow cytometry (Becton-Dickinson, Mountain View, CA, USA) using CellQuest software, and fluorescent signals were displayed as histograms.

### 2.7. RNA Extraction and Real-Time PCR

The total RNA was extracted by using a TRIzol reagent. The first-strand cDNA was synthesized from 1 *μ*g of total RNA using the Reverse Transcription System Bestar qPCR RT Kit according to the manufacturer instruction. Real-time PCR was carried out with an ABI 7500 Real-Time PCR System (Applied Biosystems, Lincoln Centre Drive, Foster City, CA 94404, USA). Each assay was performed in triplicate, and *β*-actin was used as the endogenous control gene. The relative amount of TCF21 was calculated using with a 2^−*ΔΔ*Ct^ method and normalized using GAPDH as an internal control. Primers were listed in Table 1.

### 2.8. Target Prediction and Luciferase Assay

The putative targets of miR-30-5p were predicted by the TargetScan Release 6.2. The human wild-type (WT) TCF21 was cloned. Afterwards, *TCF12* gene mutant 3′-UTR recombinant plasmid was generated using the TaKaRa MutanBEST Kit (TaKaRa, Beijing, China), which generated a mutation of 7 bps from CATTTGT to ATCACTA in the predicted miR-30-5p target binding site, identified as *TCF12*-3′ UTR-MUT. 3′ UTR sequences of TCF21 were constructed into psi-CHECK2 vector. The cells were seeded for triplicates in 24-well plates 24 hours before transfection and cotransfected with the miR-30-5p mimics or NC-mimics. 48 hours later, the cells were then harvested and lysed, the luciferase activities were performed with the Dual-Luciferase Reporter Assay System (Promega, Madison, WI, USA), and data were collected and quantitated by a3 Lumat LB 9501 luminator.

### 2.9. Plasmid Construction and Transfection

miR-30-5p mimics, inhibitor, and mock were chemically synthesized by GenePharma (Suzhou, China). Cells were seeded in 6-well or 24-well cell culture plates with fresh medium without antibiotics. Oligonucleotides were transfected into THP-1 at 80% confluence using the Lipofectamine 3000 reagent (Invitrogen, CA, USA).

The siRNA-TCF21 was obtained commercially (GenePharma). Nonsilencing siRNA acted as a negative control (NC) and was used to control any other effects of the siRNA and transfection reagents.

### 2.10. Western Blot Analysis

The Western blot assay was performed as previous description [23]. Samples were harvested and stored at −80°C. For Western blot analysis, frozen samples were sonicated on ice twice for 5 seconds in 50 mM lysis buffer (pH 7.4) containing 3.1 mM sucrose, 1 mM DTT, 10 *μ*g/ml leupeptin, 10 *μ*g/ml soybean trypsin inhibitor, 2 *μ*g/ml aprotinin, and 0.1% Triton X-100. Homogenates were centrifuged at 10000 g at 4°C for 20 minutes, and the supernatant was collected. The total protein concentration was measured using the Bradford protein assay (Bio-Rad, Hercules, CA, USA). Protein lysates (20 *μ*g) were separated using 12% SDS-PAGE and transferred to a PVDF membrane. After blocking with 5% nonfat milk, the PVDF membrane was incubated overnight with the primary antibody as follows: rabbit anti-Bcl2, rabbit anti-Bax, rabbit anti-p65, and rabbit anti-TCF21 solute in TBS-T. Membranes were washed in TBS-T (10 min × 3) and then probed with the appropriate secondary antibody (1 : 10000; Abcam). Membranes were developed using VersaDoc 5000, and band densities were measured with Quantity One 4.6 software (Bio-Rad, Hercules, CA, USA). Equal protein loading was additionally verified by measurement of the GAPDH level with rabbit polyclonal antibody.

### 2.11. Statistical Analysis

Statistical calculations were performed using Prism 6 (GraphPad Software Inc., San Diego, CA, USA). Data are presented as the mean ± standard error of the mean. Student's *t-*test was used for comparisons between two groups, and one-way or two-way analysis of variance was used for comparisons among multiple groups. Differences with *P* < 0.05 were considered as statistically significant.

## 3. Results

### 3.1. Serum Expression Level of miR-30-5p Was Downregulated in Patients with Atherosclerosis

**Figure 1:**
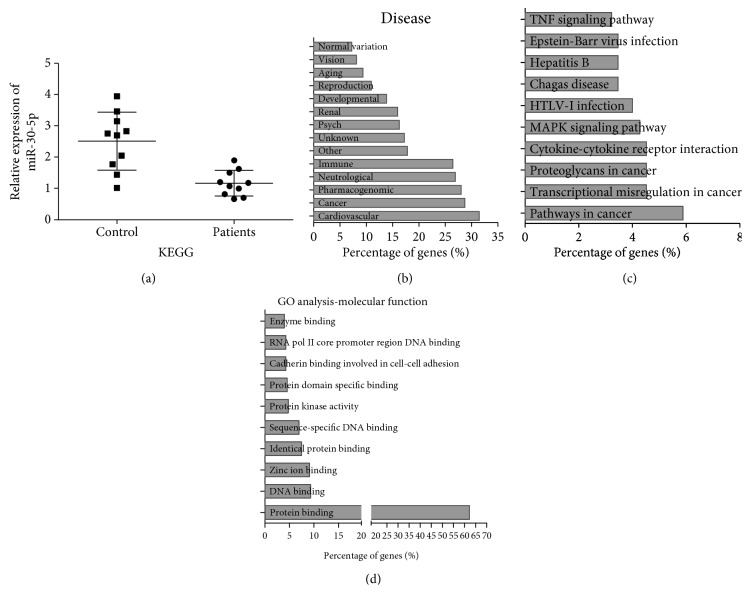
miR-30-5p was downregulated in patients with atherosclerosis and its functional enrichment analysis. (a) The expression levels of miR-30-5p in patients with atherosclerosis (patients) and normal healthy people (control) were determined by qRT-PCR. (b) GO analysis of miR-30-5p involved in different diseases. (c) KEGG analysis of miR-30-5p involved in different signal pathways. (d) Molecular function analysis of miR-30-5p by GO.

The serum expression level of miR-30-5p in patients with atherosclerosis compared to the normal group was determined by using quantitative real-time PCR (qPCR). Downregulation of miR-30-5p was found in the serum of patients with atherosclerosis in comparison to the normal group (Figure 1(a)). GO was analyzed and processed by DAVID software. GO analysis shows that miR-30-5p mainly contributed to three diseases: cardiovascular diseases (32%), cancer (28%), and neurological diseases (26%) (Figure 1(b)). Moreover, KEGG pathway analysis indicated that the TNF signaling pathway, MAPK signaling pathway, cytokine-cytokine receptor interaction, and pathways in cancer were the most significantly enriched pathways (Figure 1(c)). In terms of functional groups, protein binding accounts for 62% in molecular function of miR-30-5p (Figure 1(d)), indicating that the functional role of miR-30-5p in atherosclerosis relied on downstream target. These results showed that miR-30-5p was downregulated in atherosclerosis and highly associated with genetic profile of cardiovascular disease.

### 3.2. In Vitro Model of the Effect of miR-30-5p on Atherosclerosis

**Figure 2:**
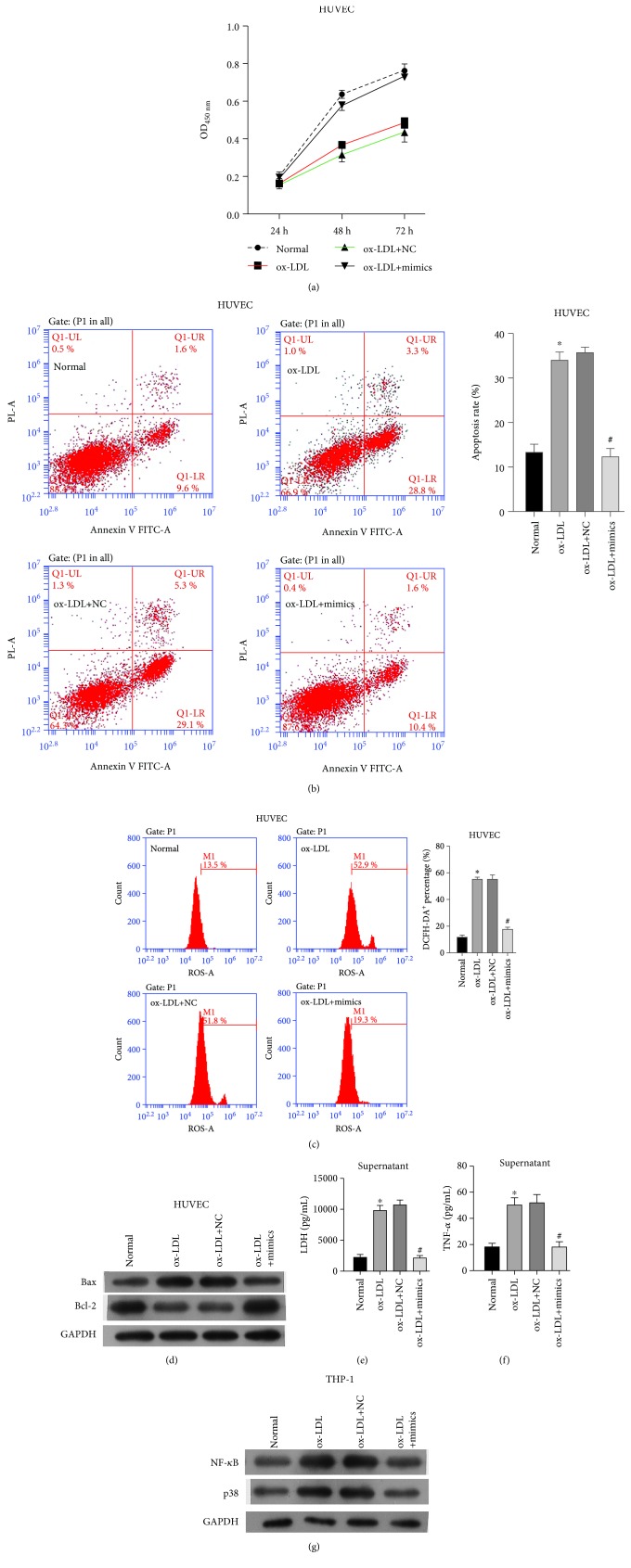
*In vitro* model of the effect of miR-30-5p on atherosclerosis. (a) Effect of miR-30-5p on the cell viability of pHUVEC cells detected by the CCK8 assay. (b) Effect of miR-30-5p on the cell apoptosis of pHUVEC cells detected by flow cytometry. (c) Effect of miR-30-5p on the ROS levels of pHUVEC cells detected by FACS. (d) Effect of miR-30-5p on the protein expression of Bax and Bcl-2. (e) Effect of miR-30-5p on the expression level of LDH detected by ELISA. (f) Effect of miR-30-5p on the TNF-*α* detected by ELISA. (g) Effect of miR-30-5p on the protein expression of NF-*κ*B and p38. ^∗^ indicated *P* < 0.05 vs. normal; ^#^ indicated *P* < 0.05 vs. ox-LDL+NC.

**Figure 3:**
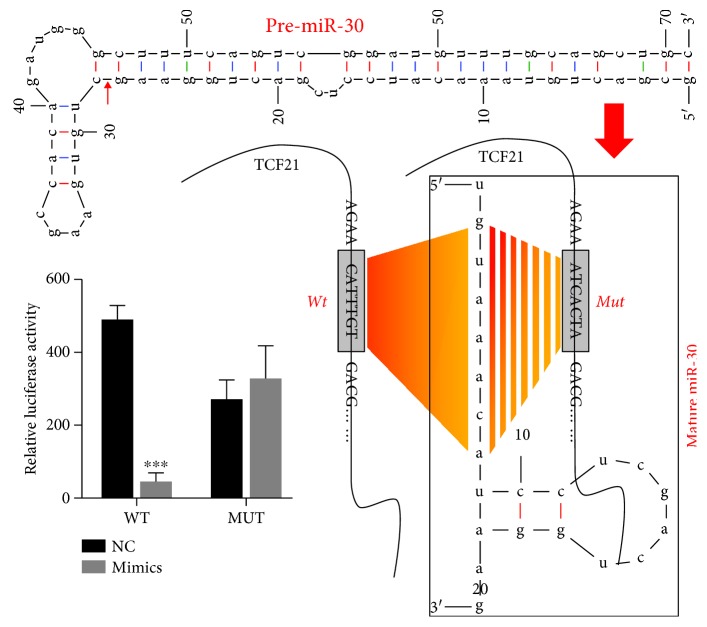
miR-30-5p directly targeted TCF21. (a) Predict binding site of miR-30-5p in TCF21. (b) Luciferase reporter assay of TCF21 3′ UTR—wild-type and mutant with miR-30-5p. ^∗^ indicated *P* < 0.05 vs. WT+NC.

In order to explore the functional role of miR-30-5p in atherosclerosis, coculturing HUVEC and the THP-1 cell model was used as the *in vitro* model simulating atherosclerosis. The efficiency of miR-30-5p mimics was evaluated by qRT-PCR, which showed significantly increased expression in comparison to that of miR-30-5p mock (NC) (data not shown). We conducted four groups of the cell model: normal cells (normal, coculturing HUVEC and THP-1 cells), ox-LDL-treated cells (blank, coculturing HUVEC and ox-LDL-treated THP-1 cells), ox-LDL-treated cells transfected with NC (NC, coculturing HUVEC and ox-LDL-treated THP-1 cells transfected with NC), and ox-LDL-treated cells transfected with miR-30-5p mimics (miR-30-5p mimics, coculturing HUVEC and ox-LDL-treated THP-1 cells transfected with miR-30-5p mimics). Firstly, the protein expression levels of TCF12 were significantly decreased in miR-30-5p mimic-transfected THP-1 cells (Figure S1). The CCK8 assay showed that ox-LDL treatment decreased the cell proliferation of HUVEC cells, while THP-1 transfected with miR-30-5p mimics protected cell proliferation in comparison to NC (Figure 2(a)). Secondly, in HUVEC cells, ox-LDL treatment promoted cell apoptosis, while miR-30-5p mimics decreased the apoptosis rate compared with NC (Figure 2(b)). Moreover, the ROS levels were markedly increased in ox-LDL condition as determined by FACS, and miR-30-5p mimics remarkably decreased ROS levels (Figure 2(c)). Proteins involved in cell apoptosis including Bax also showed increased under ox-LDL condition and decreased by miR-30-5p mimics (Figure 2(d)). However, the protein level of Bcl-2 was decreased under ox-LDL condition and increased by miR-30-5p mimics (Figure 2(d)). Lastly, the expression level of LDH was significantly increased via ox-LDL treatment while decreased in the miR-30-5p mimic group (Figure 2(e)). These results revealed that miR-30-5p mimics helped to weaken atherosclerosis through inhibiting cell apoptosis, promoting cell viability, and decreasing ROS accumulation. Consistent with that, THP-1 transfected with miR-30-5p mimics decreased the expression level of TNF-*α*, which was increased by ox-LDL (Figure 2(f)). NF-*κ*B and p38 (Figure 2(g)) were evaluated, which showed an increase by ox-LDL condition and decrease by miR-30-5p mimics. To further validate the binding ability between miR-30-5p and TCF21 (Figure 3(a)), the luciferase report system was performed to confirm that miR-30-5p can directly bind to the 3′ UTR of *TCF21* mRNA and found that miR-30-5p mimics remarkably decreased the luciferase activity of the reporter gene with wild-type TCF21 3′ UTR compared with NC-mimics (*P* < 0.001) (Figure 3(b)). These results suggested that miR-30-5p can directly bind to the 3′ UTR of TCF21 mRNA and negatively regulated the expression of TCF21 and regulated atherosclerosis through inhibition of NF-*κ*B and MAPK signal pathways.

### 3.3. In Vitro Model of the Effect of *TCF21* on Atherosclerosis

**Figure 4:**
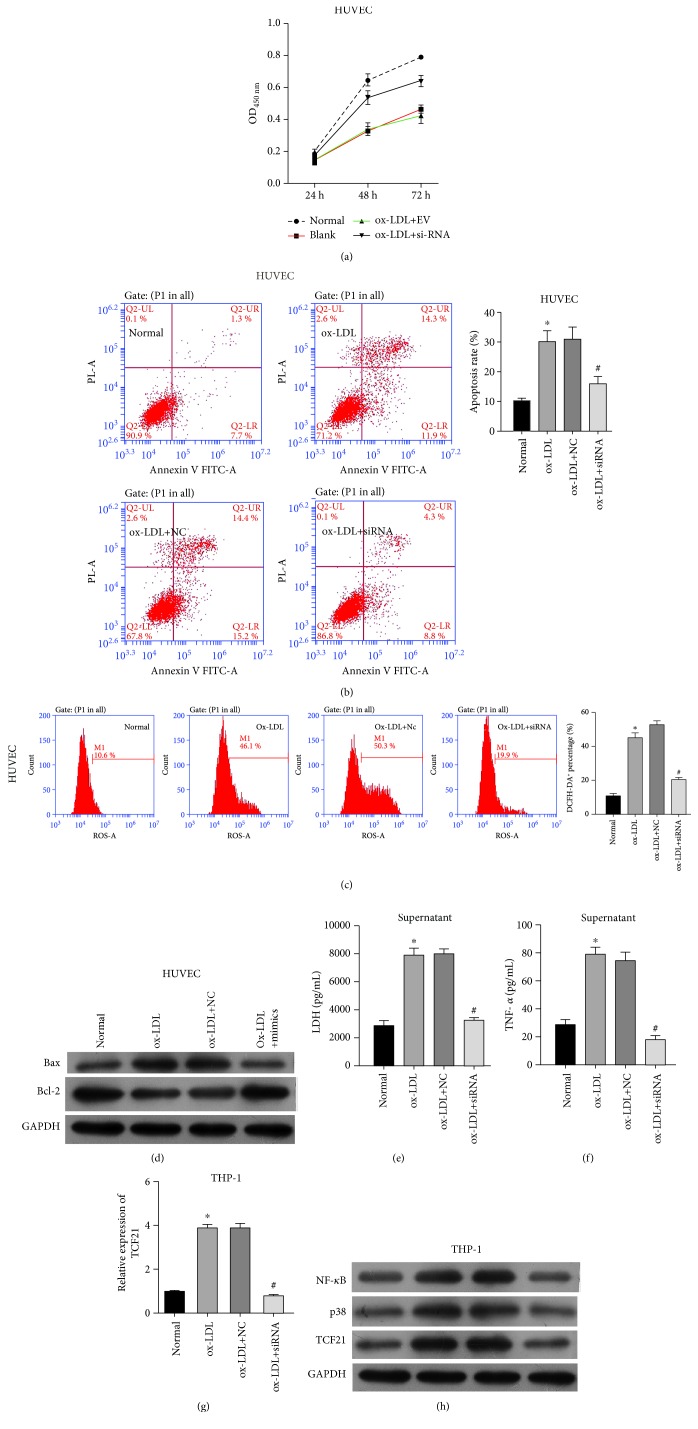
*In vitro* model of the effect of TCF21 on atherosclerosis. (a) Effect of TCF21 on the cell viability of pHUVEC cells detected by the CCK8 assay. (b) Effect of TCF21 on the cell apoptosis of pHUVEC cells detected by flow cytometry. (c) Effect of TCF21 on the ROS levels of pHUVEC cells detected by FACS. (d) Effect of TCF21 on the protein expression of Bax and Bcl-2. (e) Effect of TCF21 on the expression level of LDH detected by ELISA. (f) Effect of TCF21 on the TNF-*α* detect by ELISA. (g) Transfection efficiency of siTCF21 detected by qRT-PCR. (h) Effect of TCF21 on the protein expression of NF-*κ*B, p38, and TCF21. ^∗^ indicated *P* < 0.05 vs. normal; ^#^ indicated *P* < 0.05 vs. ox-LDL+NC.

In order to explore the functional role of TCF21 on atherosclerosis, we also conducted four groups of the cell model: normal, blank, ox-LDL-treated THP-1 cells transfected with siRNA negative control (siNC), and ox-LDL-treated THP-1 cells transfected with TCF21 siRNA (siTGF21). Firstly, the expression of TCF21 was significantly decreased in THP-1 cells after transfection of TCF21 siRNA (Figure S2). Furthermore, the CCK8 assay showed that transfection of siTGF21 protected cell proliferation in comparison to siNC and ox-LDL treatment (Figure 4(a)). Moreover, in pHUVEC cells, ox-LDL treatment promoted cell apoptosis, while siTGF21 decreased the apoptosis rate compared with siNC (Figure 4(b)). In addition, siTGF21 remarkably decreased ROS levels (Figure 4(c)), effectively alleviating oxidative stress. Cell apoptosis-related protein Bax also showed increased under ox-LDL condition and decreased by siTGF21 (Figure 4(d)). However, the protein level of Bcl-2 showed the opposite pattern of decreased under ox-LDL condition and increased by siTGF21 (Figure 4(d)). Lastly, the expression level of LDH was also significantly decreased in the siTGF21 group just as miR-30-5p mimics (Figure 4(e)). These results revealed that consistent with miR-30-5p mimics, siTCF21 also helped to weaken atherosclerosis through inhibiting cell apoptosis, promoting cell viability, and decreasing ROS accumulation. Meanwhile, THP-1 transfected with siTCF21 also decreased the expression level of TNF-*α* (Figure 4(f)) and TCF21 mRNA (Figure 4(g)), as well as TCF21 protein, NF-*κ*B, and p38 (Figure 4(h)) that were increased by ox-LDL.

### 3.4. The Effect of miR-30-5p/*TCF21* on Atherosclerosis

**Figure 5:**
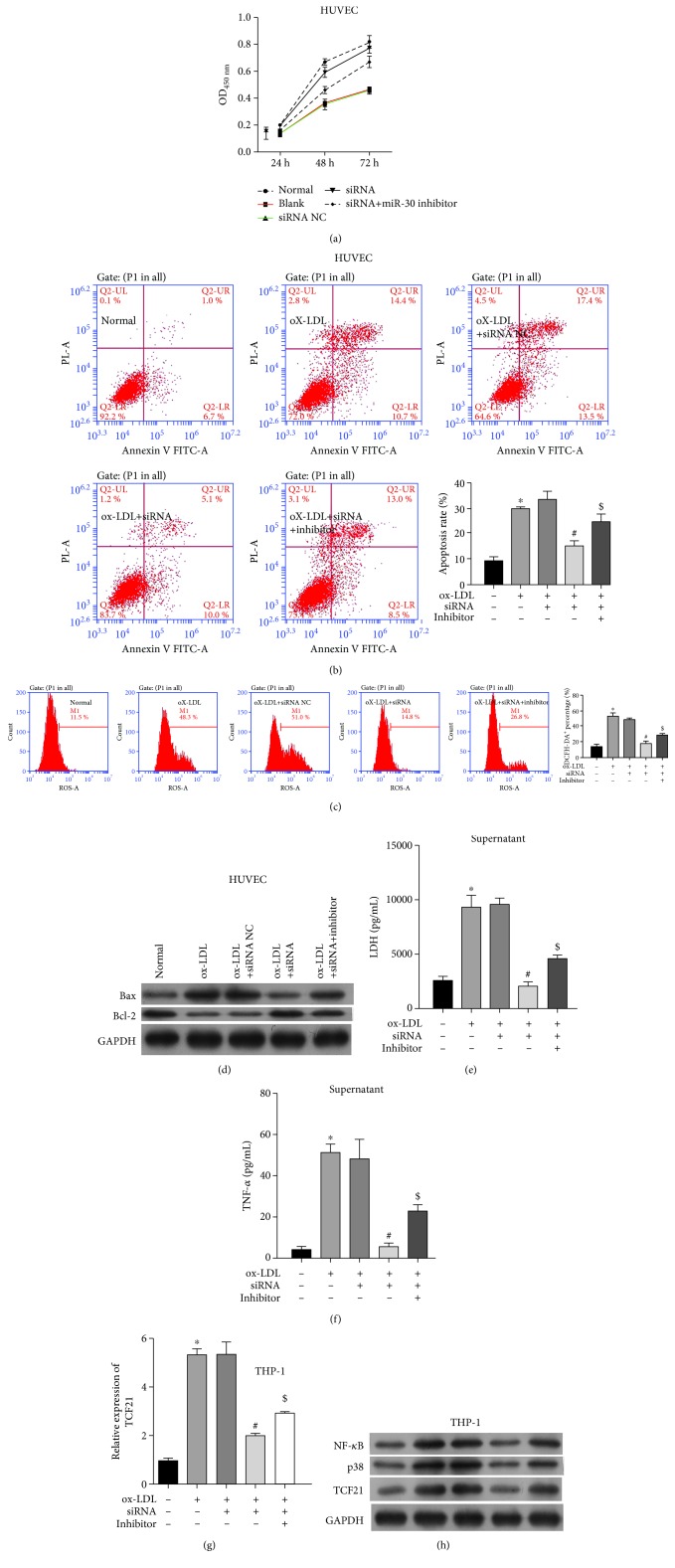
The effect of miR-30-5p/TCF21 on atherosclerosis. (a) Effect of miR-30-5p/TCF21 on the cell viability of pHUVEC cells detected by the CCK8 assay. (b) Effect of miR-30-5p/TCF21 on the cell apoptosis of pHUVEC cells detected by flow cytometry. (c) Effect of miR-30-5p/TCF21 on the ROS levels of pHUVEC cells detected by FACS. (d) Effect of miR-30-5p/TCF21 on the protein expression of Bax and Bcl-2. (e) Effect of miR-30-5p/TCF21 on the expression level of LDH detected by ELISA. (f) Effect of miR-30-5p/TCF21 on the TNF-*α* detected by ELISA. (g) Effect of miR-30-5p/TCF21 on the expression of TCF21 detected by qRT-PCR. (h) Effect of miR-30-5p/TCF21 on the protein expression of NF-*κ*B, p38, and TCF21. ^∗^ indicated *P* < 0.05 vs. normal; ^#^ indicated *P* < 0.05 vs. ox-LDL+siRNA NC; ^$^ indicated *P* < 0.05 vs. ox-LDL+siRNA.

In order to explore the functional role of miR-30-5p/TCF21 axis in atherosclerosis, five groups of the cell model were conducted: normal, blank, siNC, siTGF21, ox-LDL-treated THP-1 cells transfected with TCF21 siRNA and miR-30-5p inhibitor (siTCF21+inhibitor). Firstly, the CCK8 assay showed that addition of miR-30-5p inhibitor decreased the cell proliferation protected by siTGF21 (Figure 5(a)). Moreover, in pHUVEC cells, siTGF21 decreased the apoptosis rate, while addition of miR-30-5p inhibitor promoted cell apoptosis (Figure 5(b)). Secondly, addition of miR-30-5p inhibitor increased ROS levels decreased by siTGF21 (Figure 5(c)), effectively alleviating oxidative stress. Bax was also decreased by siTGF21 and increased by miR-30-5p inhibitor (Figure 5(d)). However, the protein level of Bcl-2 showed the opposite pattern of increased by siTGF21 and decreased by addition of miR-30-5p inhibitor (Figure 5(d)). Lastly, the expression level of LDH was also significantly decreased in the siTGF21 group while increased by addition of miR-30-5p inhibitor (Figure 5(e)). These results revealed that through inhibition of TCF21, miR-30-5p helped to protect the endothelial cell and might contribute to weaken atherosclerosis. Meanwhile, THP-1 addition transfected with miR-30-5p inhibitor increased the expression level of TNF-*α* (Figure 5(f)), TCF21 mRNA (Figure 5(g)), and TCF21 protein (Figure 5(h)) that were decreased by siTCF21. Moreover, NF-*κ*B and MAPK signal pathways involved in the regulation of miR-30-5p/TCF21 axis on atherosclerosis were detected by Western blot. Results showed that THP-1 transfected with siTCF21 and miR-30-5p inhibitor increased the expression level of NF-*κ*B and p38 (Figure 5(h)) decreased by siTCF21.

## 4. Discussion

Atherosclerosis remains the huge burden of current health care, which accounts for the major cause of myocardial infarction, stroke, and sudden death [24]. Lack of direct antiatherosclerotic therapy aimed at regression of atherosclerotic plaques remains a big challenge [25]. Therefore, development of direct antiatherosclerotic therapy should become a major goal of the modern medicine and pharmaceutical industry, considering the burden and clinical significance of the disease. Although most studies of miR-30-5p were focused on the regulation of different cancers, previous studies have shown that miR-30c could lower plasma cholesterol and atherosclerosis in mice [26]; miRNA-30c-5p could ameliorate atherosclerosis [27]. In the present study, we successfully investigated the protective role of miR-30-5p in atherosclerosis and unmasked the underlying mechanism through *in vitro* cell models. We firstly confirmed the downregulation of miR-30-5p in atherosclerosis patients, which suggested a negative regulation of atherosclerosis progression by miR-30-5p. The findings have highlighted a new functional role for tumor suppressor miRNA-30-5p in the progression of atherosclerosis, indicating that miRNA-30-5p might be a clinical marker of atherosclerosis.

Previous investigation suggested that ox-LDL, as a biomarker and therapeutic target of cardiovascular diseases [28], played an important role in plaque instability during atherosclerosis progression [29, 30]. In the present study, we constructed earlier stages (the initial damage in endothelial cells occurs) of *in vitro* atherosclerosis models with pHUVEC and THP-1 cells treated with ox-LDL. In line with a former finding that revealed downregulation of miR-30c-5p by ox-LDL [31], miR-30-5p was also downregulated by ox-LDL treatment in our study. Taking into consideration that cell death and inflammation are essential for atherosclerosis [24, 32], we firstly detect the effect of miR-30-5p on the cell apoptosis and cell viability. The relative amounts of active antiapoptotic protein (including Bcl-2 [33]) and proapoptotic protein (including Bax [34]) determined the destiny of the cells to apoptosis or not. Increased Bcl-2 accompanied by a decrease of Bax mainly functions in protecting the cells from apoptosis [35]. Our study demonstrated a reduction of Bax and a promotion of Bcl-2 and cell viability by miR-30-5p, suggesting antiapoptosis effects of miR-30-5p on atherosclerosis.

On the other hand, oxidative stress-triggered apoptosis was regulated by Bcl-2 family protein-impaired cardiomyocytes, thus facilitating for the development of cardiovascular disease [36]. We also found a reduction of the ROS level by miR-30-5p, suggesting anti-inflammation effects. Further functional assays indicated that miR-30-5p could decrease the secretion of proinflammatory cytokine TNF-*α* to modulate the pathogenesis of atherosclerosis [37]. The evaluation biomarker of myocardial injury in patients with atherosclerosis [38], LDH, was also downregulated by miR-30-5p. Generally, through antiapoptotic- and anti-inflammatory-dependent mechanisms, miR-30-5p protects against atherosclerosis.

Upon the confirmation of the important relationship between miR-30-5p and atherosclerosis, the molecular biology and signaling pathways underlying the atherosclerosis development and progression with miR-30-5p were further studied. Miller et al. reported that miR-224 interacted with the *TCF21* transcript and contributed to allelic imbalance of this gene, thus partly explaining the genetic risk for coronary heart diseases through transforming growth factor-*β* (TGF-*β*) and platelet-derived growth factor (PDGF) signaling [20]. In terms of miR-30-5p, studies have demonstrated that it mediates different factors in different pathophysiologies [39–42]. In our study, TCF21, as the downstream target of miR-30-5p confirmed by the luciferase report assay in our study, was reported to be involved in the transcriptional network linking coronary heart disease [19] and playing a crucial role in cardiac fibrosis and smooth muscle cell fate in atherosclerosis [17]. qRT-PCR, Western blot, ELISA, CCK8, and flow cytometry assays showed that the inhibitory activity of miR-30-5p against the atherosclerosis progression was achieved by regulating the TCF21-targeting NF-*κ*B and MAPK signal pathways. MAPK and NF-*κ*B signaling pathways have been reported to be involved in regulation of atherosclerosis and were considered as a potential target for treating atherosclerosis [43]. Miller et al. found that TCF21 was significantly upregulated in both asymptomatic and symptomatic atherosclerotic plaques, and the findings provide additional mechanistic insights into the *TCF21* association with respect to coronary heart disease progression [20]. Also, Nurnberg et al. found that vascular *TCF21* expression in the adult is restricted primarily to adventitial cells associated with coronary arteries and also medial SMC in the proximal aorta of a mouse. *In vitro* studies in HCASMC demonstrated that *TCF21* expression promotes proliferation and migration and inhibits SMC lineage marker expression. The data suggest that *TCF21* may have a role in regulating the differentiation state of SMC precursor cells that migrate into vascular lesions and contribute to the fibrous cap, and the *TCF21* gene contributes to coronary artery disease (CAD) risk [21]. However, Liu et al. found that in smooth muscle cells, TCF21 promotes phenotypic switching and formation of the protective fibrous cap from modulated SMCs, therefore having a protective role [44]. The results showed that TCF12 played complicated roles in atherosclerosis. Research also has shown that TCF21 interferes with the MAPK pathway to inhibit tumor growth, with interaction between specific regions of TCF21 and MAPK [45]. Meanwhile, TCF21 can directly target NF-*κ*B and MAPK signal pathways to regulate atherosclerosis. This was the first study elaborating the molecular biology and signaling pathways for miR-30-5p and atherosclerosis. Further study is required to provide direct evidences proofing the endogenous TCF21's binding to NF-*κ*B and MAPK in order to inhibit its expression.

**Figure 6:**
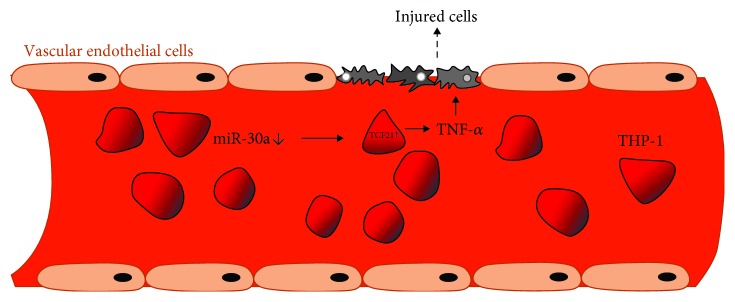
A hypothetical working model of the role of the miR-30-TCF21 axis in atherosclerosis.

To the best of our knowledge, this is the first study determining that miR-30-5p suppresses NF-*κ*B signaling and MAPK pathway-induced inflammatory responses as well as apoptosis and protects cell survival targeting TCF21 in the *in vitro* atherosclerosis model (Figure 6). Moreover, upregulation of miR-30-5p provided an efficient protective effect against the progression of atherosclerosis, providing a new insight that miR-30-5p might be a potential therapeutic target for developing a novel treatment approach against atherosclerosis.

## Figures and Tables

**Table 1:** Primers' sequences in the real-time PCR assay.

Gene	Forward primers	Reversed primers
TCF21	CCTGGCTAACGACAAATACG	TTTCAGGTCACTCTCGGGT
GAPDH	TGTTCGTCATGGGTGTGAAC	ATGGCATGGACTGTGGTCAT
miR-30-5p RT	CTCAACTGGTGTCGTGGAGTCGGCAATTCAGTTGAGACGTGAGT	
All R	CTCAACTGGTGTCGTGGA	
U6	CTCGCTTCGGCAGCACA	AACGCTTCACGAATTTGCGT

RT: reverse transcription.

## Data Availability

Data will be provided based on requirement.

## Abbreviations

KEGG: Kyoto Encyclopedia of Genes and Genomes
TCF21: Transcription factor 21
HUVEC: Human umbilical vein cell
FBS: Fetal bovine serum
ox-LDL: Oxidized low-density lipoprotein
CCK8: Cell counting kit 8.

## Supplementary Materials

Supplementary Materials Figure S1: expression of TCF21 in THP-1 cells without ox-LDL treatment. A: expression of miR-30-5p was validated using qRT-PCR. B: expression of TCF21 was measured using Western blot. ^∗∗^ indicated *P* < 0.01 vs. NC. Figure S2: expression of TCF21 in THP-1 cells without ox-LDL inducing. A: effect of siRNA targeting TCF21 was confirmed using qPCR (A) and Western blot. ^∗∗^ indicated *P* < 0.01 vs. NC.
