# Corrigendum to “MicroRNA-30-5p Suppresses Inflammatory Factor-Induced Endothelial Cell Injury by Targeting TCF21”

**DOI:** 10.1155/2021/9816785

**Published:** 2021-10-22

**Authors:** Zhenyu Zhou, Yu Chen, Dongying Zhang, Shiyong Wu, Tao Liu, Guoqiang Cai, Shu Qin

**Affiliations:** ^1^Department of Cardiology, The First Affiliated Hospital of Chongqing Medical University, Chongqing 400016, China; ^2^Department of Cardiology, Nanchong Central Hospital, The Second Clinical School of North Sichuan Medical College, Nanchong, China; ^3^Comprehensive Ward, Nanchong Central Hospital, The Second Clinical School of North Sichuan Medical College, Nanchong, China

In the article titled “MicroRNA-30-3p Suppresses Inflammatory Factor-Induced Endothelial Cell Injury by Targeting TCF21” [1], the authors identified an error introduced during the preparation of the manuscript in Figure 4, where the GAPDH bands were duplicated between Figures 4(d) and 4(h). In Figure 4(d), “ox-LDL + mimics” should also be corrected to “ox-LDL + siRNA.”

Additionally, the significance indicators were omitted from Figures 1, 2, 4, and 5 in error, and in Table 1, a primer was not included. The corrected figures and tables are as below.

Due to an error during manuscript preparation, the incorrect miRNA was stated in the title of the manuscript. The article has been updated from “MicroRNA-30-3p” to “MicroRNA-30-5p” as shown above.

The authors apologize for these errors and confirm that they do not impact the conclusions of the article.

## Figures and Tables

**Figure 1:**
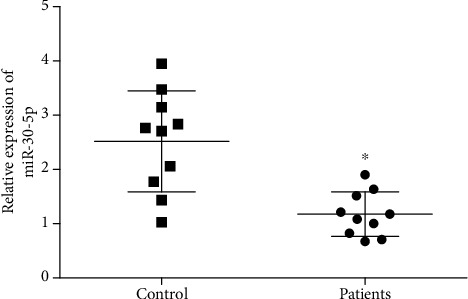
miR-30-5p was down-regulated in patients with atherosclerosis and its functional enrichment analysis. (a) The expression levels of miR-30-5p in patients with atherosclerosis (patients) and normal healthy people (control) were determined by qRT-PCR. ∗indicated p <0.05 vs. control.

**Figure 2:**
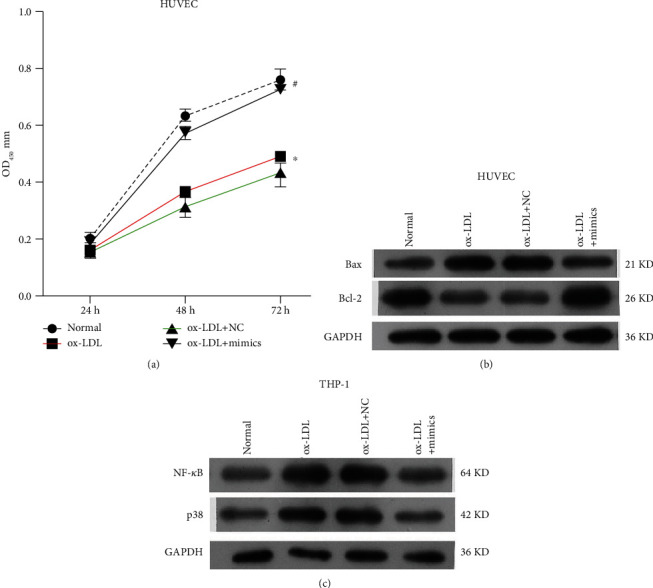


**Figure 3:**
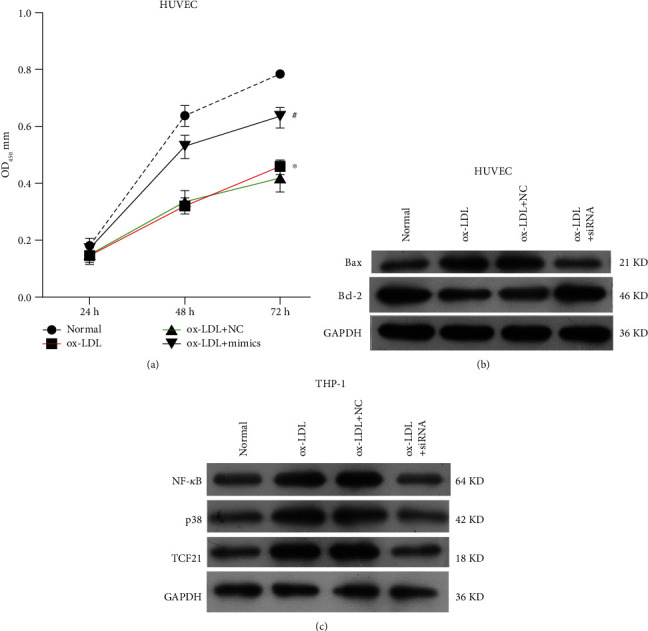


**Figure 4:**
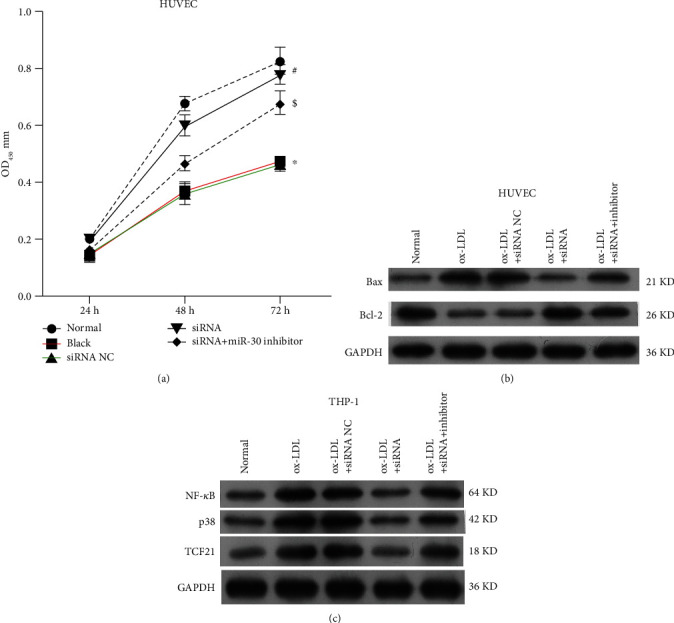


**Table 1:** Primers' sequences in the real-time PCR assay.

Gene	Forward primers	Reversed primers
TCF 21	CCTGGCTAACGACAAATACG	TTTCAGGTCACTCTCGGGT
GAPDH	TGTTCGTCATGGGTGTGAAC	ATGGCATGGACTGTGGTCAT
miR-30-5p RT	CTCAACTGGTGTCGTGGAGTCGGCAATTCAGTTGAGACGTGAGT	
miR-30-5p F	ACACTCCAGCTGGGTGTAAACATCCTACACT	
All R	CTCAACTGGTGTCGTGGA	
U6	CTCGCTTCGGCAGCACA	AACGCTTCACGAATTTGCGT
