# Supplementary material for: MicroRNA-30-3p Suppresses Inflammatory Factor-Induced Endothelial Cell Injury by Targeting TCF21
Source: Mediators Inflamm. 2019 Jul 2;2019:1342190. doi: 10.1155/2019/1342190 (PMC6636441; doi:10.1155/2019/1342190)
Supplement: Supplementary Materials — Figure S1: expression of TCF21 in THP-1 cells without ox-LDL treatment. A: expression of miR-30-5p was validated using qRT-PCR. B: expression of TCF21 was measured using Western blot. ∗∗ indicated P < 0.01 vs. NC. Figure S2: expression of TCF21 in THP-1 cells without ox-LDL inducing. A: effect of siRNA targeting TCF21 was confirmed using qPCR (A) and Western blot. ∗∗ indicated P < 0.01 vs. NC. [file 1342190.f1.pdf]

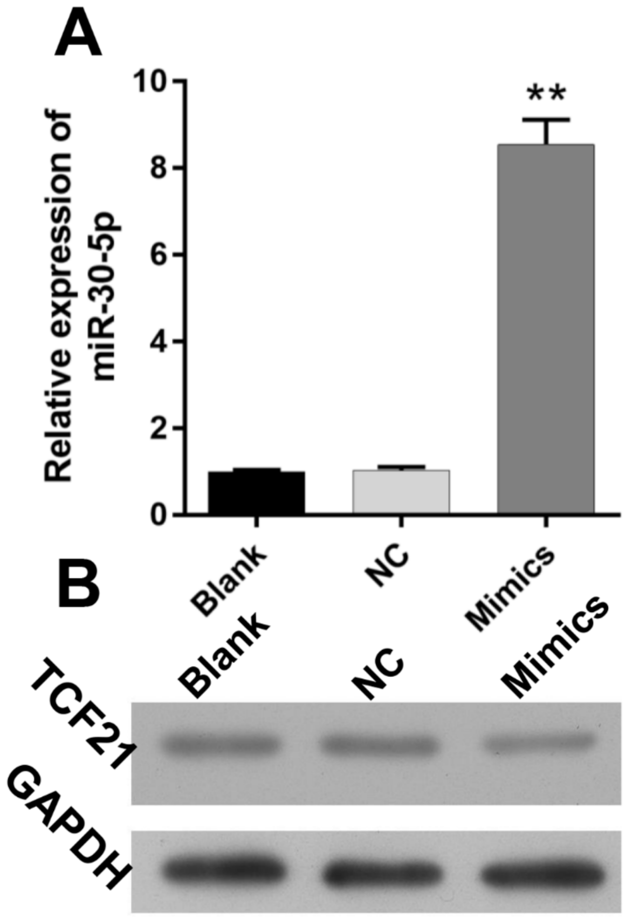

Figure S1. Expression of TCF21 in THP-1 cells without ox-LDL treatment. A. expression of miR-30-5p was validated using qRT-PCR. B. expression of TCF21 was measured using western blot. **indicated p<0.01 vs. NC.

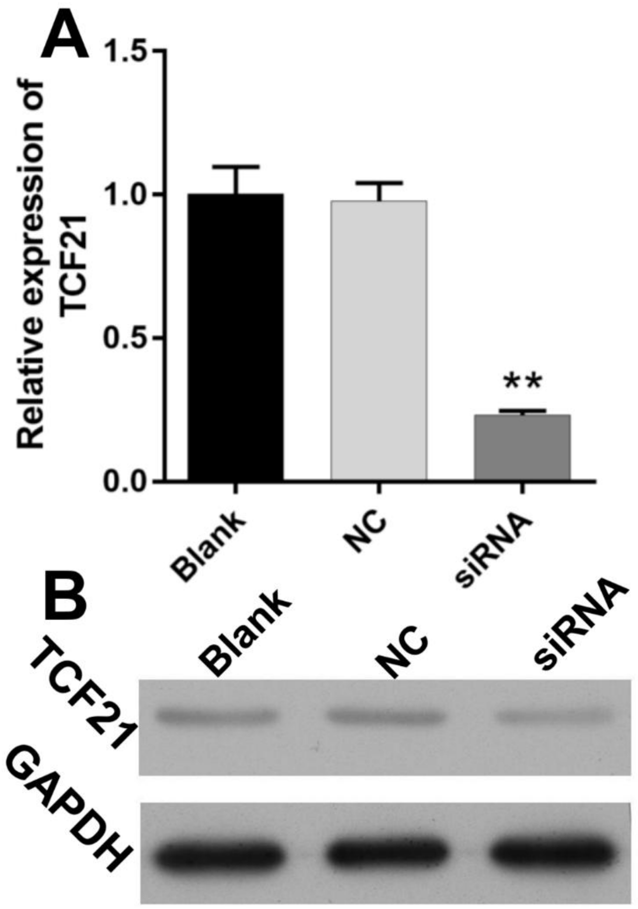

Figure S2. Expression of TCF21 in THP-1 cells without ox-LDL inducing. A. Effect of siRNA targeting TCF21 was confirmed using qPCR (A) and Western blot. **indicated $p<0.01$ vs. NC.
